# Attitudes Towards Bilingualism: Insights From Parents of Children With Down Syndrome

**DOI:** 10.1111/1460-6984.70153

**Published:** 2025-11-08

**Authors:** Rebecca Ward, Eloi Puig‐Mayenco

**Affiliations:** ^1^ Department of Psychology University of South Wales, UK; ^2^ School of Education, Communication and Society King's College London, UK

**Keywords:** bilingualism, bilingual experience, clinical practice, down syndrome, multilingualism, practitioner guidance, professional advice

## Abstract

**Background:**

Recent research indicates that bilingualism does not exacerbate the language learning difficulties commonly seen in children with down syndrome (DS). However, misconceptions persist about its potential negative impact.

**Aims:**

This study aimed to investigate the attitudes, perceptions and experiences of bilingual parents or guardians raising children with DS, focusing on challenges, strategies, and professional advice received. To our knowledge, this is the first study to explore this topic specifically within this population.

**Methods and Procedures:**

Fifty‐seven parents participated in an online survey exploring these factors using both quantitative and qualitative responses. A significant majority of participants (87.72%) had chosen to raise their children bilingually, motivated by cultural heritage, community support and perceived cognitive benefits.

**Outcomes and Results:**

Overall, parental attitudes toward bilingualism were positive. Many reported receiving supportive information yet concerns and a lack of professional support were prevalent. Challenges included a shortage of bilingual therapists, inadequate educational provisions, and insufficient tailored information on bilingualism. Professional advice varied substantially. Speech and language therapists generally provided positive advice, while social workers, health visitors, and psychologists offered mixed recommendations. Nursery and school practitioners often exhibited uncertainty about bilingualism, contributing to parental concerns. Despite this, children with DS exposed to multiple languages were observed to be acquiring both languages with expected language patterns observed. Just over half (52%) of the families had concerns about raising their child bilingually, primarily about the difficulty of learning two languages, lack of support, and concerns around confusion. Crucially, those receiving positive professional advice were more likely to continue promoting bilingualism.

**Conclusions and Implications:**

This study highlights the need for consistent, evidence‐based support for families raising bilingual children with DS. Improved professional training and resources are needed to ensure children with DS can receive equitable access to bilingualism and are able to thrive in bilingual environments.

**WHAT THIS PAPER ADDS:**

*What is already known on this subject*

- Research indicates that bilingualism does not hinder language development in children with down syndrome (DS), whilst research into bilingualism highlights possible cognitive and social benefits. Studies show that whilst raising bilingual children, maintaining both languages supports cultural identity and engagement. Research into the advice given to parents of children with developmental disorders in general suggest that parents may receive inconsistent advice from professionals, leading to uncertainty about bilingual development. However, to date little literature has explored this within the context of DS and studies exploring parents’ experiences specifically has gained little attention.

*What this paper adds to the existing knowledge*

- This study highlights how a child's diagnosis influences parental decisions about bilingualism, often leading to uncertainty and a lack of support. Many parents hope bilingualism will enhance their child's communication and access to a variety of opportunities, yet they frequently receive no or conflicting guidance. However, parents of younger children report more positive advice, indicating a potential shift in professional perceptions. Crucially, the advice parents receive directly impacts their decisions on language use. These findings emphasise the need for clear, evidence‐based professional guidance to support bilingual families, ensuring consistency in recommendations across professional groups and providing confidence in understanding bilingual development for children with DS.

*What are the potential or actual clinical implications of this study?*

- In the UK, the Royal College of Speech and Language Therapists (RCSLT) recognise that bilingualism does not cause speech or language disorders and should be actively supported. Professionals are encouraged to advise families to maintain home language use, fostering communication with families, encouraging social development, and cultural identity. However, in this study parental reports reveal inconsistencies in advice, across practitioner groups with many receiving mixed or no guidance. This highlights gaps in the understanding of bilingualism in DS and the need for further guidelines, especially for GPs and paediatricians. Further research is needed to explore professionals' perspectives and improve bilingual support for children with DS as well as longitudinal studies and research within different contexts.

## 1 Introduction

Bilingualism serves not only as a means of communication but as a strong link to cultural identity and familial bonds (Curdt‐Christiansen and Huang 2020; Peace‐Hughes et al. 2021). Globally, the prevalence of bilingualism is rising, with over half of the world's population using more than one language daily (Grosjean 2010, 2021). At the same time, bilingualism is increasingly recognised as having a positive impact on social, cognitive and economic outcomes. Parental attitudes toward bilingualism^1^ are likely to be influenced by some of these factors as well as their individual circumstances. These attitudes toward bilingualism can significantly influence whether families choose to maintain their home language(s) or introduce their child to a second language (Hollebeke et al. 2023; Surrain 2021).

In contexts with limited support for bilingualism, concerns about bilingual parenting are widespread. For example, Meisel (2019) highlights worries parents have about bilingualism, such as cognitive overload and confusion from exposure to multiple languages. Parents also worry about children's social integration, particularly early proficiency in the societal language (Sevinç 2022), fearing academic disadvantages. These concerns illustrate the complexity of bilingual parenting in environments that do not fully embrace linguistic diversity, emphasising the need to address parental anxieties and ensure children benefit from bilingual contexts.

Despite efforts to address these misconceptions, significant gaps persist in understanding parental perspectives across diverse bilingual contexts (King 2016). For families where a child is born with a developmental disability, as in the case with children with down syndrome (DS), this decision‐making process becomes more complex. As delays and challenges with language acquisition are considered a hallmark of DS, questions have arisen as to what impact bilingualism will have, with notable apprehension about raising them in multilingual environments (Howard et al. 2021; Kay Raining‐Bird et al. 2012). The aim of the present study is to elucidate caregivers’ diverse experiences of raising a bilingual child with DS, including concerns, support from professionals and strategies for success. By exploring these perspectives, we can better inform policies and practices that support bilingual development and enrich the linguistic and cultural experiences of children growing up in diverse linguistic environments.

## 2 Literature Review

### 2.1 Language Development, DS and Bilingualism

As the most common chromosomal disability, DS is characterised by several distinct challenges and differences in physical, cognitive and linguistic development. Although there is substantial inter‐individual variation, a reoccurring finding is that delays and difficulties with linguistic skills are not fully accounted for by the observed intellectual difficulties. Language acquisition is thus seen as an area that poses substantial challenges for children with DS. In addition, some aspects of linguistic development are found to be particularly challenging, such as expressive morphosyntax and phonological development (Roberts et al. 2007; Smith et al. 2020). Speech intelligibility can also be affected due to anatomical differences and motor planning issues, such as hypotonia affecting oral muscles (Kent and Vorperian 2013). On the other hand, studies reporting strengths in visual processing and receptive skills have been used as the basis for interventions with this population, which are reported to be successful across development including adolescence and adulthood (Sepúlveda et al. 2013; Smith et al. 2020; van Bysterveldt et al. 2006).

Whilst difficulties with language development are a specific concern for children with DS, questions have followed regarding the ability of these children to acquire two (or more) languages. This is often the case for children with a range of language difficulties. For example, Kay‐Raining Bird and colleagues (2012) conducted a parental survey exploring experiences of bilingualism amongst caregivers of children with autism and found that practitioners did not always support the view that these children could or should be raised bilingually. In addition, lack of services and support around bilingualism was a concern for these families. In contrast to this view, emerging research, indicates that bilingualism does not impede the linguistic development for children with DS, as discussed below (Cleave et al. 2014; Kay‐Raining Bird et al. 2005; Ward and Sanoudaki 2021, 2024).

### 2.2 Perspectives Toward Bilingualism

Perspectives on bilingualism vary considerably, and these are likely to be influenced by perceptions of how bilingualism might impact development for those with language‐learning difficulties. These perspectives also depend on cultural, societal and linguistic contexts with parental perspectives often reflecting broader societal attitudes toward bilingualism, which can differ greatly depending on the linguistic context. Taking the UK as an example, in England, where English is the predominant language, bilingualism might be viewed with more scepticism and caution compared to Wales, where both Welsh and English hold official status, and bilingualism is encouraged and more culturally supported (Howard et al. 2021). In Wales, the bilingual context is further supported by institutional and societal structures that promote the use of both Welsh and English.

Across all parts of the UK, linguistic diversity is prominent with the 2021 Census (Office for National Statistics) revealing significant multilingual communities, particularly in urban areas such as London, Manchester and Birmingham. Languages such as Polish, Romanian, Punjabi and Urdu are widely spoken alongside English, reflecting the multicultural nature of these cities. Despite this, societal support for bilingualism varies, and parental decisions about bilingualism can also be influenced by the availability of resources, societal attitudes, and the perceived benefits for their children. Whilst for some bilingual up bringing can be a result of parental decisions, the necessity of bilingualism has also been highlighted by Grosjean (2010), among others. This means that for some families, bilingualism is a natural part of their lives rather than something that can be opted for or removed.

For children with DS, language decisions require careful consideration due to observed language delays. Some have feared bilingualism could cause confusion or further delays (Kay‐Raining Bird et al. 2012; de Valenzuela et al. 2016). However, as noted above, research shows bilingualism does not hinder linguistic development in DS (Cleave et al. 2014; Kay‐Raining Bird et al. 2005, 2016; Ward and Sanoudaki 2021, 2024) or other neurodevelopmental conditions like autism (Reetzke et al. 2015; Siyambalapitiya et al. 2022; Zhou et al. 2019). As an example, Ward and Sanoudaki (2021) found that bilingual children with DS had comparable language abilities to monolingual peers in the shared language (English). Studies also show that bilingual language development in DS follows similar patterns to monolinguals, suggesting children can thrive in bilingual environments with proper support (Kay‐Raining Bird et al. 2005; Ward and Sanoudaki 2021, 2024).

In contrast to these concerns, studies in the field suggest that in fact bilingualism may enhance certain cognitive abilities, namely, executive functioning, inhibitory control and perspective taking (Montgomery et al. 2022; Peristeri et al. 2021a, 2021b). Bilingualism might also serve as a protective mechanism against age‐related cognitive decline (Venugopal et al. 2024; though note the inconsistencies across studies in this field). However, the fear of language delays and confusion means that many families may opt for monolingual approach (Hampton et al. 2017; Kay‐Raining Bird et al. 2012; Yu 2009). For example, Hampton et al. (2017) conducted interviews with parents of autistic children and parents of typically developing (TD) children. Both groups expressed common concerns about bilingualism and recognized various societal and cultural factors that influenced their choice to provide a bilingual language environment for their children. However, parents of autistic children identified additional factors unique to their situation. For example, they believed that bilingualism might interact with the challenges associated with autism, potentially impacting not only language development but also cognitive and behavioural development. These concerns were heightened for autistic children with more significant language difficulties.

Similarly, Kay‐Raining Bird et al. (2012) conducted a survey of parents of autistic children to explore the decisions made surrounding bilingualism and the concerns that they had. Many parents reported that professionals did not support their decision to raise their children bilingually. Concerns were raised about the lack of specific support and services tailored to bilingual children and their families, alongside more general concerns about their child's capacity to be bilingual. To date, no study has explored parents’ perspectives toward bilingualism for children with DS specifically.

### 2.3 Professional Perspectives and Clinical Guidelines

Professional attitudes toward bilingualism, particularly among those working with children who have developmental disabilities, also vary widely. An online international study by Marinova‐Todd et al. (2016a) aimed to examine professionals’ practices and opinions toward bilingualism for children with developmental disabilities specifically and found that while some professionals supported bilingualism, viewing it as having cognitive and social benefits, others express concerns, leading to inconsistent advice for families. In addition to this mixed attitude toward bilingualism, what practitioners reported should be available for these children did not often align with practices and actual support available.

A more recent study by Sharpe and Perovic (2024) explored speech and language therapists (SLTs) views of bilingualism for children with speech, language and communication needs, comparing perspectives between the UK and Singapore. They found that although there was variability in therapists’ opinions and practices, generally SLTs held attitudes in line with recent evidence (i.e., bilingualism does not cause or contribute to language delays or difficulties). Only a small proportion of therapists reported giving advice against bilingual exposure, and these were more likely to be based in Singapore rather than the UK. This study concluded by expressing the need for more substantial training around bilingualism, as well as an awareness of how to work with bilingual families in an evidence‐based manner.

As professionals are often the first source of advice for parents, the uncertainty and inconsistency could result in increased stress and anxiety in relation to how to best support their child and whether raising them bilingually is the best approach (Davis et al. 2023; Howard et al. 2021; Sevinç 2022). This is concerning, not only because evidence suggests that bilingualism is possible for these children, but also given the growing evidence that bilingualism can strengthen family bonds and support cultural identity, which are crucial for the well‐being of children with developmental disabilities (Sevinç 2022; Digard et al. 2023).

These differing views toward bilingualism are likely to influence practices within education and healthcare, though specific professional guidelines exist in some cases for bilingualism. Most notably, the Royal College of Speech and Language Therapists (RCSLT; the professional body for speech and language therapists in the UK) offer set guidelines that SLTs should follow when working with bilingual children and families, as well as offering a bespoke bilingualism training module (Royal College of Speech and Language Therapists 2019a; 2019b Pert and Bradley 2018). Furthermore, this guidance states that bilingual children and their families should receive an equitable service which may include interpreters and additional therapy resources.

Specific guidance is provided for all aspects of assessment and intervention for speech and language needs which is based on recent evidence in the field (Royal College of Speech and Language Therapists, 2019a, 2020). For example, this guidance states that assessments should be carried out in the home language, case histories should include detailed information about language exposure and use, and that the overall outcome of SLT should be for the child to develop or maintain their home language. This guidance and support that professionals provide for families are likely to be very influential in caregivers’ decision‐making around bilingualism, particularly if they feel uncertain about the outcome of bilingual exposure and bilingual interventions.

Finally, the United Nations Convention on the Rights of the Child (UNCRC; 1989) also outline the rights of children and young people with disabilities. These international guidelines stipulate that children have the ‘right to use his own language,’ whereby a ‘child belonging to a linguistic minority group shall not be denied’ this right. This closely aligns with the UK Equality Act (2010) which provides statutory requirements in relation to ensuring that those with a protected characteristic, such as a disability, are not treated unfairly on this basis. Therefore, removing a child's access to bilingualism due to a disability such as DS, is not only contradictory to the current evidence base, but could also be seen as treating them with a lack of equality. These legal requirements and guidelines may also therefore impact both practitioners’ and caregivers’ perspectives toward bilingualism for this population.

## 3 Aims and Research Questions

Given this backdrop, the aim of the present study is to further understand the perspectives, experiences and concerns surrounding bilingualism for parents or guardians of children with DS. Current best practice and clinical guidelines state that bilingualism should be considered as an advantage (Royal College of Speech and Language Therapists 2019a; 2019b). At the same time, studies suggest that there are variations in how this works in practice and that caregivers may be advised to adopt a forced monolingualism approach whereby the family is directed toward focussing on one language only (Kay‐Raining Bird et al. 2012; de Valenzuela et al. 2016). The current study therefore also aims to investigate whether practices in advising and supporting bilingual families of children with DS have become more aligned with evidence‐based practice. Specifically, the study will address the following research questions:

1. What decisions do parents or guardians of children with DS make around language use and what influences their decision making?
2. How do parents or guardians of children with DS perceive the challenges of raising their child(ren) in a bilingual environment and what strategies do they use to overcome these challenges?
3. How does the advice that parents of bilingual children with DS receive from practitioners influence their decisions about language use?
4. Does the context of bilingualism affect the decision‐making process and perspectives of parents or guardians of children with DS?

### 3.1 Methods

The present study adopted an online cross‐sectional survey targeting parents who have a child with DS. The survey was accessible via Qualtrics, where participants were provided information about the aims and objectives of the research and of the inclusion criteria. Responses were collected from June 2023 to April 2024. Ethical approval was granted from Swansea University (Application Reference 2 2023 5983 5469) and King's College London (Application Reference MRA‐22/23‐35450).

### 3.2 Participants

Recruitment of participants was conducted via direct contact with organisations who support children with DS and their families, direct emails to relevant schools and social media advertisements. In total, eighty‐one participants were recruited. After removing duplicate responses and incomplete responses, the final sample included 57 parents who met the full inclusion criteria. These criteria stipulated that participants must be (1) parents or guardians of a child with DS and (2) had either chosen to raise their child bi/multilingually or had considered this as an option. Participants were predominantly mothers (89.29%) with the remaining participants being the father (10.71%) of a child with DS.

The average age of the parents was 44.64 (SD = 5.79), with the children's average age of 111.43 months (9 years 3 months; SD = 111.38). Geographically, the majority of respondents resided in the UK with most from England (*n* = 30, 52.63%), Wales (*n* = 12, 21.05%) or Scotland (*n* = 3, 5.26%). Others lived in Switzerland (*n* = 7, 12.28%) France (*n* = 2, 3.51%), Brazil (*n* = 1, 1.75%), Finland (*n* = 1, 1.75%) and Nigeria (*n* = 1, 1.75%). Two children were reported to also have a diagnosis of Autism, and one had a co‐occurring diagnosis of ADHD. Most either had no hearing disorders (*n* = 38, 66.67%) or had a corrected hearing disorder (*n* = 15, 26.31%). In terms of birth order, most were first born (*n* = 29, 50.88%) or second born (*n* = 17, 29.83%). Further demographic information can be found in Table 1.

**TABLE 1:** Demographic information about the participants and their children.

Caregiver's relationship to child	
Mother	51 (89.47)
Father	6 (10.53)
Caregiver's age (years)	
Mean	44.52
SD	5.81
Child's age (months)	
Mean	110.09
SD	110.88
DS subtype	
Trisomy 21	49 (85.97)
Mosaic	3 (5.26)
Translocation	1 (1.75)
Unaware	4 (7.02)
Hearing disorder	
None	38 (66.67)
Corrected	15 (26.31)
Uncorrected	4 (7.02)
Currently attending SLT	
Yes	43 (75.44)
No	14 (24.56)
Caregiver's highest level of education	
PhD/Doctorate	2 (3.51)
Masters	25 (43.86)
Bachelors	20 (35.09)
BTEC	3 (5.26)
NVQ	2 (3.51)
AS/A Levels	3 (5.26)
Entry Level/GCSE	1 (1.75)

*Notes*: Total number of participants is presented with % in parenthesis, unless otherwise specified.

For education level, note that one participant noted that they had a degree from a different country that was not comparable with these qualifications.

A total of 24 languages were spoken by the participants and their families with the majority speaking English (*n* = 51, 96.23%)^1^, Welsh (*n* = 8, 15.09%) and Spanish (*n* = 8, 15.09%). Information regarding the language backgrounds of the participants and the second primary caregiver are reported in Table 2.

**TABLE 2:** Language background information for the participants and the child's second parent or guardian.

Parent/Guardian 1	Excellent	Good	Average	Poor	Very poor
English skills					
Speaking	41 (71.93)	12 (21.53)	3 (5.26)	0 (0.00)	1 (1.75)
Writing	39 (69.42)	14 (24.56)	3 (5.26)	0 (0.00)	1 (1.75)
Reading	42 (73.68)	13 (22.81)	1 (1.75)	0 (0.00)	1 (1.75)
Understanding	40 (70.18)	14 (24.56)	2 (3.51)	0 (0.00)	1 (1.75)
Second language skills					
Speaking	28 (49.12)	14 (24.56)	8 (14.04)	3 (5.26)	1 (1.75)
Writing	26 45.61)	8 (14.04)	12 (21.53)	3 (5.26)	5 (8.77)
Reading	31 (54.39)	8 (14.04)	9 (15.79)	3 (5.26)	3 (5.26)
Understanding	32 (56.14)	14 (24.56)	7 (12.28)	1 (1.75)	0 (0.00)

Parent/Guardian 2					
English skills					
Speaking	37 (64.91)	9 (15.79)	2 (3.51)	1 (1.75)	1 (1.75)
Writing	34 (59.65)	11 (19.23)	3 (5.26)	1 (1.75)	2 (3.51)
Reading	34 (59.65)	12 (21.05)	2 (3.51)	1 (1.75)	1 (1.75)
Understanding	34 (59.65)	11 (19.23)	2 (3.51)	0 (0.00)	1 (1.75)
Second language skills					
Speaking	26 (45.61)	6 (10.53)	3 (5.26)	5 (8.77)	2 (3.51)
Writing	25 (43.86)	4 (7.02)	2 (3.51)	6 (10.53)	5 (8.77)
Reading	25 (43.86)	6 (10.53)	4 (7.02)	5 (8.77)	2 (3.51)
Understanding	29 (50.88)	5 (8.77)	4 (7.02)	3 (5.26)	1 (1.75)

*Note*: Total number of participants is reported for each category, with % in parenthesis.

### 3.3 Materials

#### 3.3.1 Questionnaire

The questionnaire was designed by consulting previously published papers exploring attitudes toward bilingualism in different populations (Kay‐Raining Bird 2012; Sharpe and Perovic 2024), as well as addressing key questions needed to answer the research questions outlined above. The final questionnaire contained 72 closed questions (demographics = 5; child's information = 9; parent/guardian 1 = 13; parent/guardian 2 = 14; language background = 10; attitudes toward bilingualism = 10; Advice = 10; impact of COVID = 1) and two open ended questions, one addressing any impact that COVID‐19 had on their decision making and one asking parents to share any further information that they felt was of relevance. The questionnaire took between 20 and 25 minutes to complete. The questionnaire is provided in the Supporting Information.

### 3.4 Results

**FIGURE 1:**
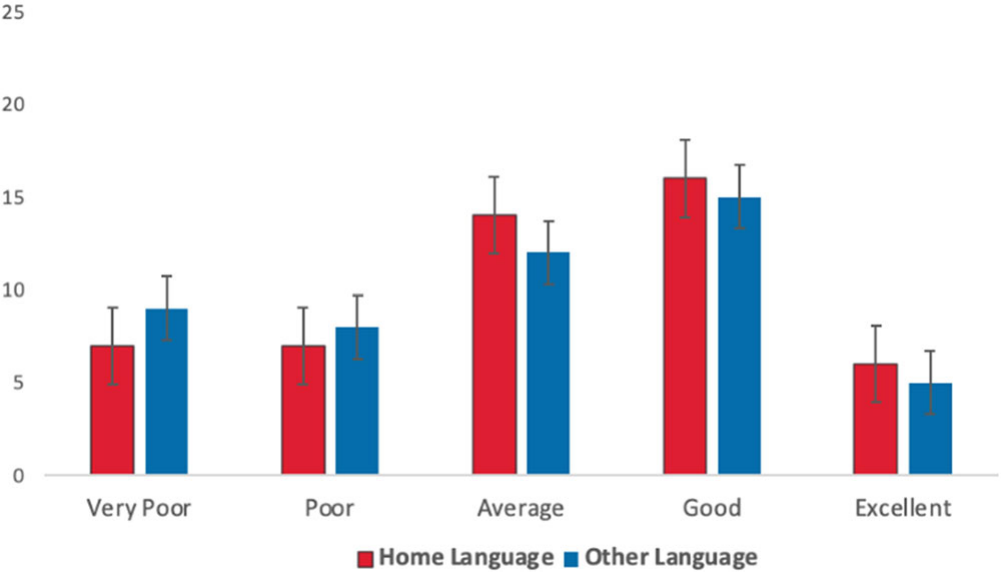
Parent‐reported receptive language skills for their children in English and their other language.

**FIGURE 2:**
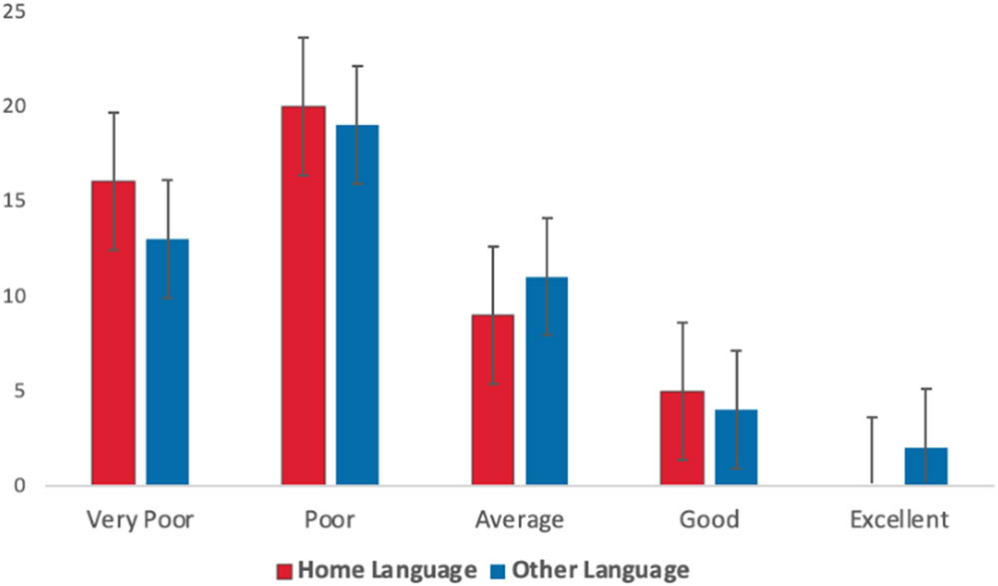
Parent‐reported expressive language skills for their children in English and their other language.

Firstly, information was gathered about the children's language exposure, language use and expressive and receptive language skills in all their languages, as shown in Table 3, Figures 1 and 2.

**TABLE 3:** Children's language exposure and proficiency.

**English AoE**					
Mean	4.38				
SD	22.33				
**Other language AoE**					
Mean	5.5				
SD	25.37				
**English skills**	**Excellent**	**Good**	**Average**	**Poor**	**Very poor**
Expressive	1 (1.75)	6 (10.53)	12 (21.05)	24 (42.11)	14 (24.56)
Receptive	5 (8.77)	18 (31.58)	16 (28.07)	11 (19.30)	7 (12.28)
**Other language skills**	**Excellent**	**Good**	**Average**	**Poor**	**Very poor**
Expressive	1 (1.75)	3 (5.26)	11 (19.30)	22 (38.60)	17 (29.83)
Receptive	6 (10.53)	13 (22.81)	15 (26.32)	10 (17.54)	10 (17.54)

*Notes*: Age is reported in months. For language proficiency, total number of participants is reported for each category, with % in parenthesis, unless otherwise specified.

Abbreviation: AoE, age of exposure.

Overall, a similar pattern was found for children's home language and their other language in that expressive skills were weaker than receptive skills, a finding that mirrors previous literature (Næss et al. 2011). A Wilcoxon signed‐rank test showed that this difference between expressive and receptive abilities was statistically significant in both languages (English *z* = –4.331, *p* < 0.001, other language *z* = –4.372, *p* < 0.001). Additionally, there was no significant difference between children's home language and their other language for either, receptive *z* = –1.060, *p* = 0.275 or expressive skills, *z* = –1.071, *p *= 0.276. This suggests that these bilingual children were managing both languages without a clear disadvantage on the development of either language, aligning with previous literature (Kay Raining Bird et al. 2005; Ward and Sanoudaki 2021, 2024).

### 3.5 What Decisions Do Parents of Children With DS Make Around Language Use and What Influences Their Decision Making?

**FIGURE 3:**
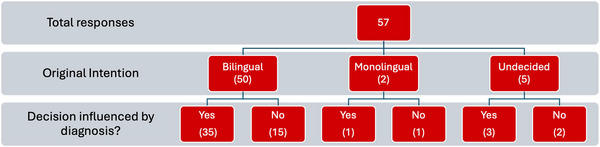
A flowchart summarising the initial intentions of families and the extent to which diagnosis of DS affected their decisions.

The primary aim of the questionnaire was to identify the extent to which parents or caregivers chose to raise their children with DS bilingually, and whether this choice aligned with their practices for other siblings without DS. Participants were asked whether they intended to raise their child with DS bilingually before the child's birth. Of the respondents, 50 (87.72%) indicated that they intended to raise their child bilingually, five (8.77%) were undecided and two (3.51%) had decided in favour of a monolingual approach. When subsequently asked whether the DS diagnosis influenced their decision‐making, the majority (35 of the 50 families initially planning bilingualism) reported that the diagnosis did influence their final decision (70.00%), whereas 15 (30.00%) maintained their original plan. A flowchart summarising these decision pathways is provided in Figure 3.

**FIGURE 4:**
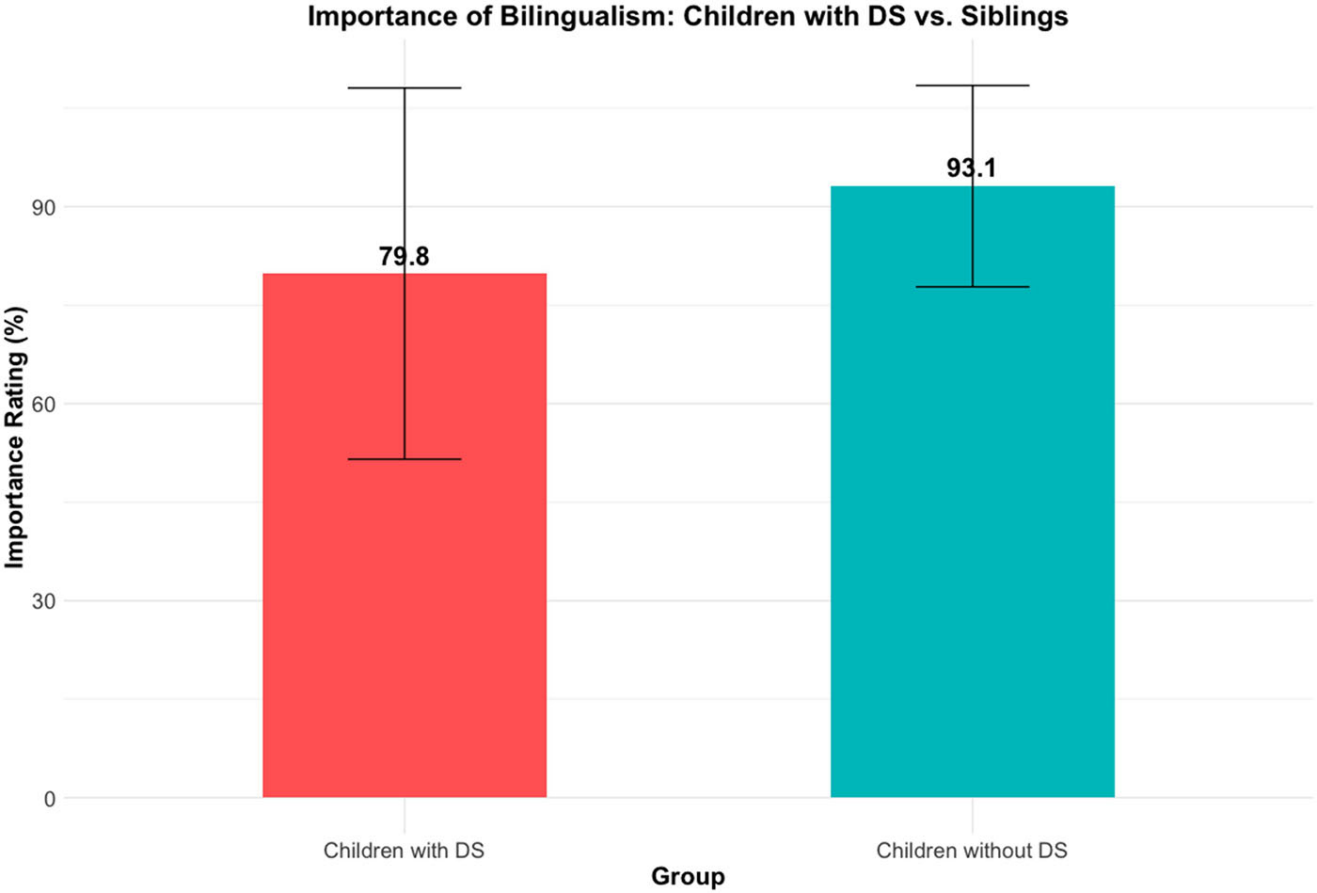
Bar chart comparing the perceived importance of bilingualism ratings for children with DS compared to their siblings.

After examining intentions, we explored the extent to which families considered bilingualism important. Results showed that overall, participants thought bilingualism was a strong asset to their children with and without DS. However, there was a difference between the perceived importance for children with DS compared to their siblings without DS. In households with siblings (*n* = 39), respondents rated the importance of bilingualism for their children with DS at a mean of 79.81% (SD = 28.26), compared to a mean of 93.12% (SD = 15.33) for their children without DS. A paired‐sample *t*‐test revealed that this difference was statistically significant, *t*(48) = –3.68, *p* < 0.001, with a mean difference of –12.71% (95% CI [–19.65, –5.78]). These findings indicate that, although bilingualism was valued across the board, families consistently rated its importance higher for children without DS (see Figure 4).

The investigation of actual language exposure practices revealed that most participants were raising their children bilingually or multilingually (*n* = 50, 87.72%). The majority used a second language in the home environment (*n* = 53, 92.98%). In contrast, 5.26% (*n* = 3) chose to expose their child only to the societal language, while 12.28% (*n* = 7) provided more detailed explanations of their language use. Of these, two reported that they focused exclusively on the societal language, with only limited exposure to the home language. Another two respondents indicated that they used the societal language alongside Makaton or British Sign Language (BSL), opting to limit the use of the home language. One participant noted that living in a bilingual environment left them with no choice but to use both languages. The remaining two respondents reported that their children were growing up in a trilingual setting: one family limited exposure to two languages after diagnosis, while the other encouraged trilingualism.

In relation to the choice when it came to siblings with DS, the responses showed that out of the 57 families, 39 reported siblings in the household and all of them reported choosing bilingualism with siblings. Although similar patterns emerged for siblings—with all 39 families reporting bilingual practices—the overall high rate of bilingual choices should be interpreted with caution, as families with a favourable attitude toward bilingualism may have been more likely to participate. To minimise this bias, efforts were made to ensure that the call for participants explicitly targeted families and households where bilingualism was a possibility, including those who may have ultimately chosen not to pursue it.

**FIGURE 5:**
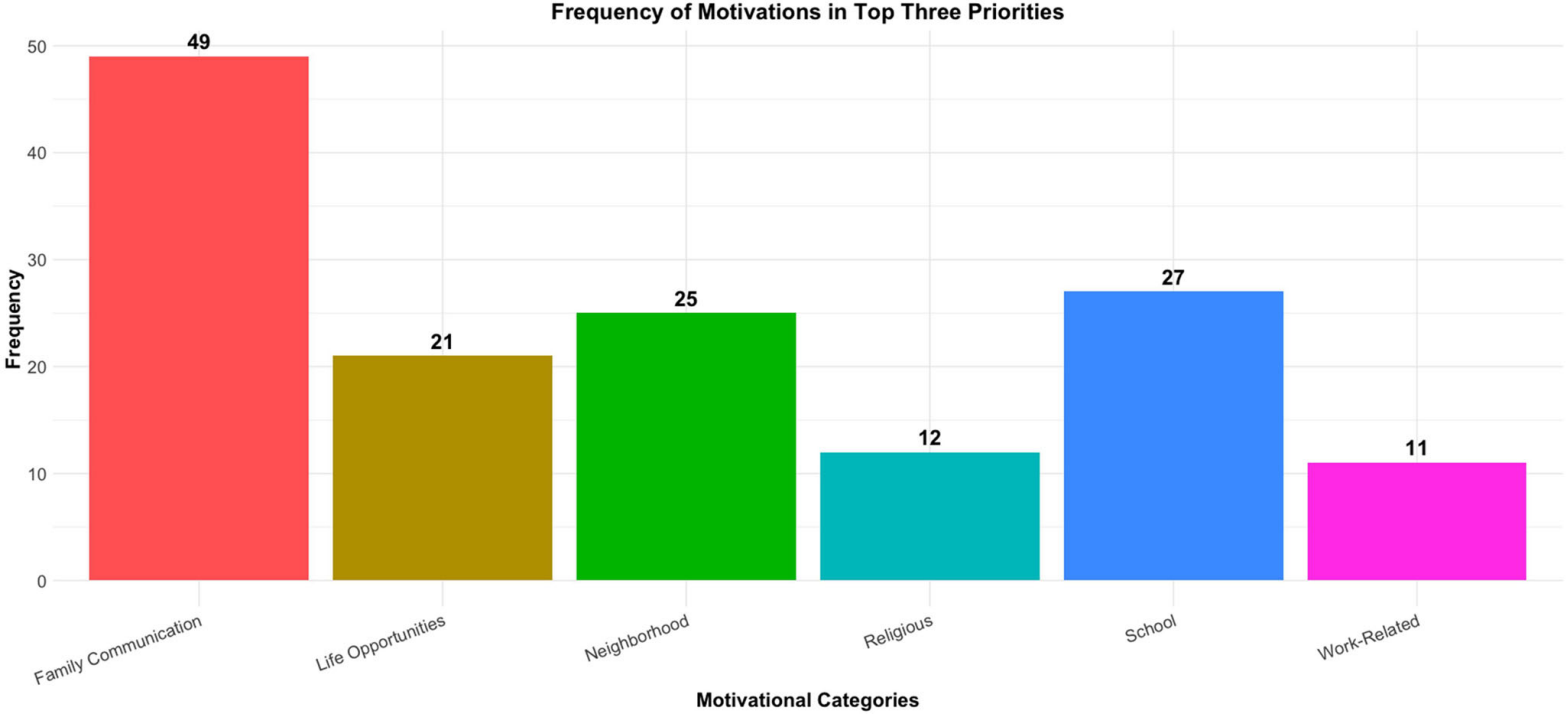
Parents' motivations for choosing to raise their child bilingually.

Participants also ranked their motivations for choosing bilingualism. Communication with family members emerged as the predominant factor. Out of the 57 respondents, 42 identified family communication as their top priority, while five ranked it as their second priority, and two placed it as their third (see Figure 5). Communication with the local neighbourhood and at school was also important; for example, 17 respondents ranked local neighbourhood communication as a second priority and seven as a third priority, while 27 respondents listed school communication among their top three priorities (with 20 specifically ranking it third).

Additionally, 21 respondents considered bilingualism's potential to offer life opportunities as a priority, with one placing it first, 13 second and nine third. In contrast, fewer respondents viewed religious (*n* = 12) or work‐related reasons (*n* = 11) as key motivations, as these factors rarely appeared in their top three priorities. In addition to ranking their priorities, participants had the opportunity to elaborate on other reasons for choosing bilingualism.

### 3.6 How Do Parents or Guardians of Children With DS Perceive the Challenges of Raising Their Child(ren) in a Bilingual Environment and What Strategies Do They Use to Overcome These Challenges?

**FIGURE 6:**
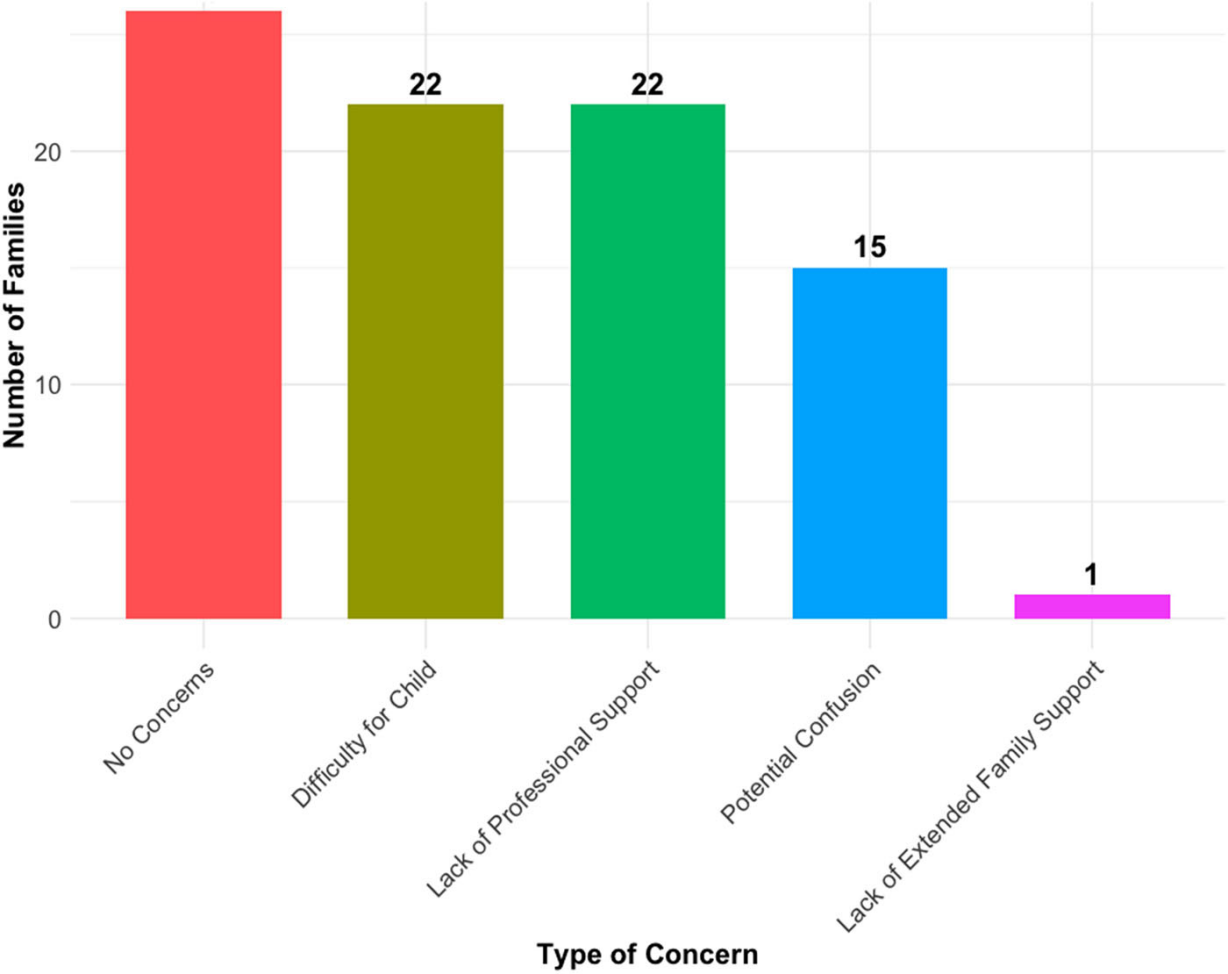
Concerns reported by parents about raising their child bilingually.

We further investigated whether families had concerns regarding the bilingual upbringing of their children. Responses were nearly evenly split. Twenty‐seven families (47.37%) reported no concerns, while 30 families (52.63%) expressed varying levels of concern. Among those with concerns, 22 families (39.28%) indicated that the potential difficulty of bilingualism for their child was a major worry appearing as one of their top three concerns, and an equal number cited the lack of professional support for bilingualism as a significant concern (39.28%). Furthermore, 15 families (26.78%) were worried about potential confusion arising from bilingual exposure, and one family noted a lack of extended family support. An illustration of this is presented in Figure 6.

Despite these concerns, the rate of opting for a bilingual upbringing was high, as noted earlier. We then explored the strategies families use to support bilingualism. Among the 46 families who responded, the most commonly reported primary strategy was teaching language skills at home, selected by 32 families (69.6%). The second most used primary strategy was receiving bilingual speech–language pathology support, reported by five families (10.9%), followed by having different people speak different languages at home, chosen by three families (6.5%). For secondary strategies, 42 families provided responses. The most frequently mentioned was having different people speak different languages at home, reported by 18 families (42.9%). The second most common was speaking different languages at different times in the home, used by 10 families (23.8%), followed by teaching language skills at home as a secondary method, reported by six families (14.3%). Among the 38 families who provided a tertiary strategy, the most frequently mentioned approach was speaking different languages at different times at home, reported by 12 families (31.6%). Watching TV in two or more languages was the second most common strategy, used by 10 families (26.3%), followed by attending preschool or school in a second language, reported by eight families (21.1%).

These findings suggest that, despite concerns, families employ various proactive strategies to support bilingualism in children with DS. The high rate of home‐based language teaching highlights a strong parental commitment to fostering bilingual development through daily interaction and intentional language exposure. Additionally, the use of structured supports, such as bilingual speech–language therapy, language‐focused schooling and media in multiple languages, demonstrates that families are actively seeking out both informal and formal resources to reinforce bilingual skills across settings.

In addition to identifying the strategies used, families were asked to reflect on how effective they perceived these strategies to be in promoting bilingualism in their child with DS. Although only a small number of families rated their efforts as extremely successful (three families, 6.5%) or not at all successful (three families, 6.5%), the majority fell somewhere in between. Nearly half of the respondents (23 families, 50%) reported their strategies as somewhat successful, while 11 families (23.9%) described them as successful, and six families (13%) considered them very successful.

### 3.7 How Does the Advice That Parents of Bilingual Children With DS Receive From Practitioners Influence Their Decisions About Language Use?

Finally, advice may (in)directly affect the (un)conscious decisions parents make in relation to the upbringing of their children. Given the influence that professional guidance can have, we explored the extent to which families received advice regarding bilingualism. The practitioners examined in this study include those who are often in contact with families of young children, particularly those with developmental conditions. These include nursery, primary, and secondary education staff; speech and language therapists or pathologists; paediatricians or general practitioners; health visitors; social workers; and psychologists. These professionals are commonly engaged in supporting early development and could therefore play a key role in shaping parental attitudes and decisions around bilingual language use in the home. We first asked about advice received from any professional, before asking specific questions about specific professionals that families may have come into contact with.

When asked about advice received from educational professionals (such as teachers or nursery staff), of the 55 families who responded to this section, the majority (25 families, 45.5%) reported receiving no advice on bilingualism. A smaller group (16 families, 29.1%) recalled being positively encouraged to raise their child bilingually. In contrast, three families (5.5%) reported being explicitly advised not to raise their child bilingually, while four families (7.3%) noted that the practitioners expressed uncertainty or ambivalence about bilingualism. An additional six families (10.9%) provided responses categorized as ‘other/not applicable.’ For these, no further information was provided. Only one family (1.8%) reported being given specific guidance to use each language at different times. These findings suggest that while supportive advice was present in some cases, a substantial number of families received either no guidance or ambiguous messages, highlighting a potential gap in educational support for bilingualism in children with DS.

When asked about advice received from SLTs, just under half (23 families, 41.8%) reported being encouraged by SLTs to raise their child bilingually. However, a significant portion, 18 families (32.7%) said they received no advice on the topic. Four families (7.3%) were explicitly advised against bilingualism, while six families (10.9%) indicated that the SLT was unsure or ambivalent about the issue. Two families (3.6%) selected ‘other/not applicable,’ and two families reported receiving specific strategy‐based advice: one was told to use each language at different times, and another to use each language with different people. These findings suggest that SLTs play a more proactive and supportive role compared to staff in educational settings when it comes to bilingualism, but they also reveal a degree of inconsistency in the guidance provided, which indicates a need for clearer, evidence‐based recommendations in this area.

Advice received from paediatricians and health visitors showed a similar pattern, with most families reporting limited or no guidance on bilingualism. Out of the families who responded, 29 (52.7%) indicated that they received no advice from paediatricians, while 31 (56.4%) reported the same for health visitors. Positive encouragement to raise their child bilingually was reported by 15 families (27.3%) for paediatricians and 10 families (18.2%) for health visitors. A small number of families were explicitly advised not to raise their child bilingually—two in the case of paediatricians (3.6%) and one for health visitors (1.8%). Additionally, some practitioners expressed uncertainty about the topic (four families for paediatricians, 7.3%, six for health visitors, 10.9%), and some other families gave responses that fell into the ‘other/not applicable’ category (five and seven families, 9.1% and 12.7% respectively).

A similar proportion of psychologists were reported to provide guidance on bilingualism. Of the families who responded, 30 (54.5%) reported receiving no advice from psychologists on the matter. Nine families (16.4%) indicated that they were encouraged to raise their child bilingually, while only one family (1.8%) reported being advised against it. A small number of families noted that psychologists expressed uncertainty (two families, 3.6%), and 13 families (23.6%) gave responses categorized as ‘other/not applicable’. These numbers suggest that psychologists are not commonly involved in conversations about bilingualism or may not feel equipped to advise on it. This points to a broader trend across professional roles of limited, inconsistent support for families navigating bilingual decisions for children with DS.

**FIGURE 7:**
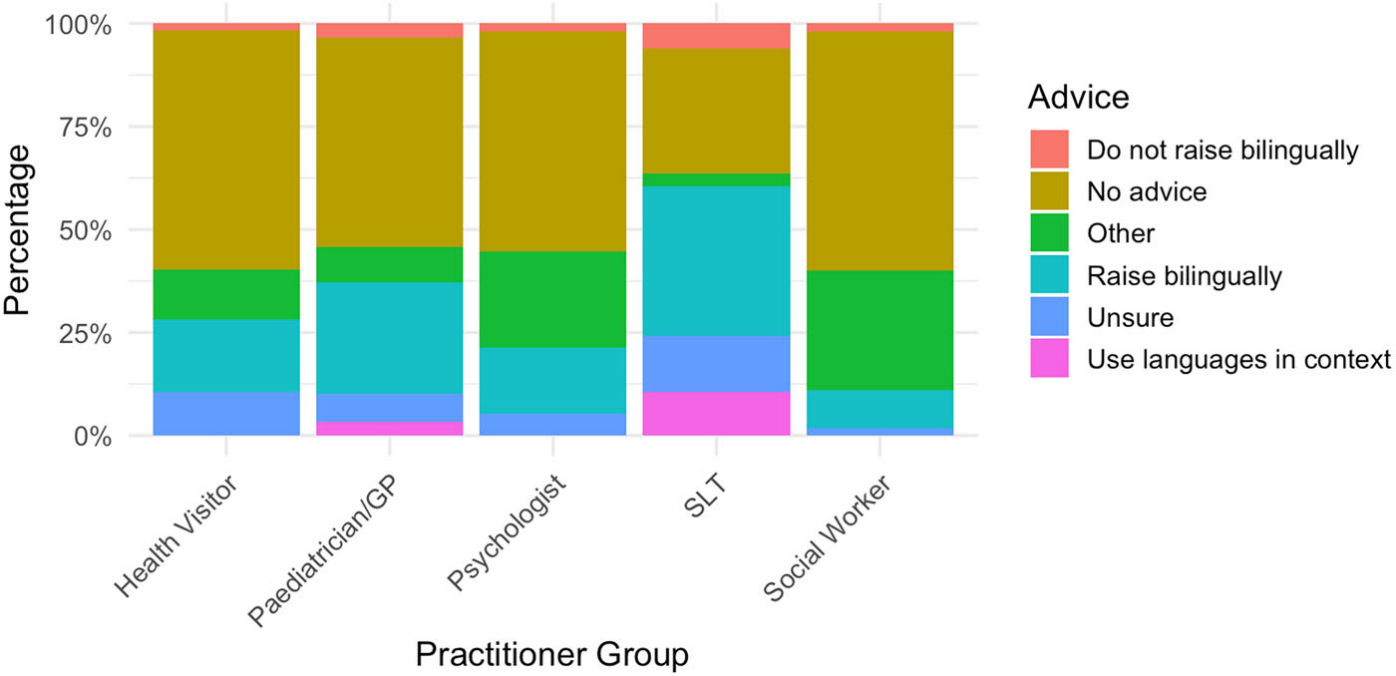
Advice given separated by practitioner group.

The patterns of advice reported across practitioner groups reveal considerable variability in both the frequency and content of guidance provided to families. Although some professionals—such as SLTs—were more likely to offer supportive advice, others, including health visitors and psychologists, were less frequently engaged in discussions about bilingualism. These findings are summarised and visually represented in Figure 7, which illustrates the distribution of advice received from each professional group.

Finally, due to changes in the professional guidance for SLTs and the developing research in this field, a binary logistic regression was conducted to examine whether child age (in months) predicted the likelihood of parents reporting that they had received positive or negative advice about bilingualism. Note that for this analysis, we coded mixed advice or unsure as being negative as this type of advice did not explicitly support bilingualism. Those receiving no advice were not included in this analysis. The overall model was statistically significant, *χ^2^* (53) = 4.32, *p* = 0.038, but explained a very small proportion of the variance (McFadden *R* ^2^ = 0.011, i.e., age explained only 1.1% of the variance in positive/negative advice). Child age was not a significant individual predictor, *p* = 0.130 and the odds ratio was 0.990, indicating that each additional month of age was associated with a 1% decrease in the odds of receiving positive advice (i.e., parents of older children were more likely to receive negative advice), although this effect did not reach statistical significance.

### 3.8 Does the Context of Bilingualism Affect the Decision‐Making Process, Perspectives of Parents or Guardians of Children With DS and Perceived Outcomes of Bilingualism?

To explore parental perspectives on bilingualism for children with DS further, we compared the nature and consistency of professional advice across four distinct contexts: Wales, England, Switzerland and Other. This was decided based on the original aims of the study, these differing linguistic environments, but was also influenced by the number of responses from Switzerland which was not expected. Although we could have excluded these responses as Switzerland was not an initial target in the study, we felt it was important to include them to represent a highly multilingual context. This allowed us to explore how contextual factors, such as policies, cultural expectations and access to support services shape parental attitudes and decision‐making processes. For example, Wales is an officially bilingual country and while the number of bilingual speakers is relatively low (e.g., Welsh‐English bilingual speakers 17.8%–19%; Office for National Statistics 2021), there is generally a lot of promotion for bilingualism and opportunities to learn the official language (Welsh). In contrast, England is also a multilingual and multicultural country; however, there is less official recognition and support for speakers of other languages. Switzerland shares the support and promotion of bilingualism; however, the number of bilingual and multilingual speakers is far higher than in Wales (68%; Federal Statistical Office 2019). Those families residing in countries other than Wales, England or Switzerland were grouped as ‘Other.’ A summary of the descriptive statistics relating to these locations is provided in Table 4.

**TABLE 4:** Descriptive statistics across countries: Perceived importance, and receipt of professional advice.

	Wales	England	Switzerland	Other
**Importance (%)**				
Mean	88.58	83.00	78.00	71.38
SD	20.61	26.91	33.66	32.31
**Proportion of advice (%)**				
Yes	8.33	24.14	33.33	37.50
No	91.67	75.86	66.67	62.50

Firstly, an ANOVA was conducted to compare the parents’ perceptions of the importance of bilingualism across the locations. This revealed no significant differences between regions both for the children with DS, *F*(3, 53) = 0.69, *p* = 0.559, or their siblings without DS *F*(3, 47) = 1.11, *p* = 0.355. Overall, parents and guardians consistently rated bilingualism as having a high importance, with similar levels of importance regardless of geographical context, indicating that parental decisions about raising their children bilingually are driven by broader considerations such as access to resources, and individual family circumstances rather than location‐specific influences.

**FIGURE 8:**
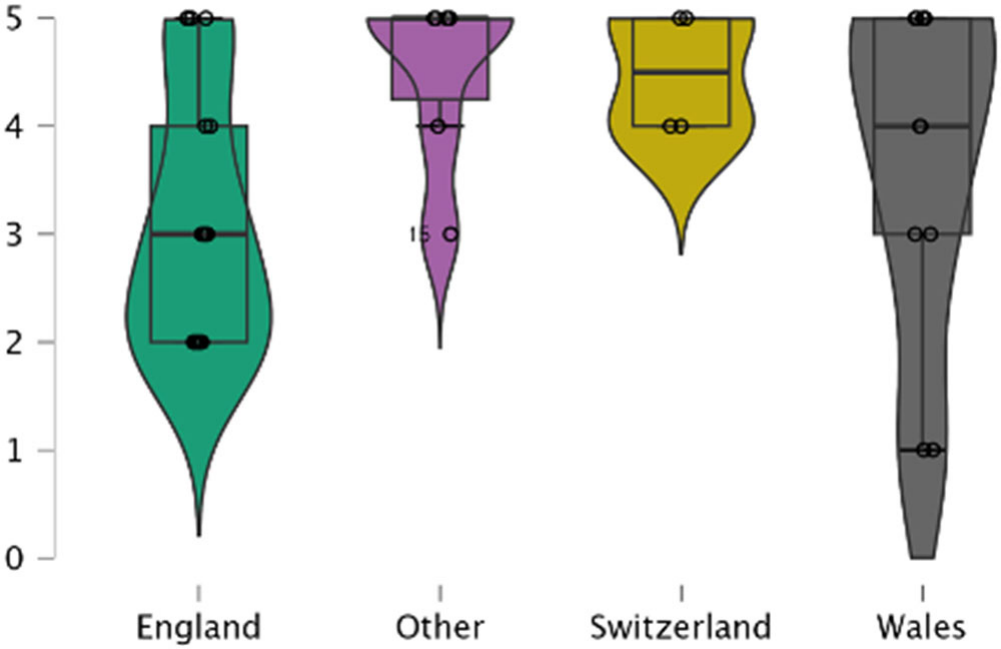
Parent reported success rates in raising their child with DS bilingually.

A Kruskal–Wallis test was then conducted to examine differences in parental self‐reported success in raising their children bilingually across Wales, England, Switzerland and Other. The results indicated no significant differences among the four groups, *H*(3) = 4.833, *p* = 0.184, suggesting that regional context did not have a statistically meaningful impact on perceived success in bilingual upbringing. Although the test did not detect significant differences, descriptive trends suggest that parents in England reported comparatively lower success rates in success with bilingualism, and more variability in Wales (see Figure 8). This observation warrants further research.

Finally, a Chi‐square test of independence was conducted to examine whether the likelihood of receiving professional advice regarding bilingual language exposure differed across the four countries. The results indicated no significant association between country and receiving of professional advice, *χ^2^* (3) = 2.725, *p* = 0.436, suggesting that access to such advice did not vary meaningfully across geographical contexts. This consistency across regions indicates a broadly shared approach among practitioners in advising families on bilingual language development for parents of children with DS, though caution is warranted with interpreting these findings due to the small number of families from some locations.

### 3.9 Parental Reflections on Bilingualism and DS

In the final section, parents were provided with an opportunity to share any further thoughts or reflections on their experience of raising bilingual children with DS. These qualitative responses are summarised according to the key themes below:

#### 3.9.1 Parental Decision‐Making

Several parents expressed that bilingualism was an important aspect of their child's upbringing, even considering their child's diagnosis and language‐related difficulties. On the whole, families reported remaining committed to bilingualism as a way to offer more opportunities, despite their concerns.

*‘Many people told me not to burden my son with DS with two languages. I am glad I did not listen to them. Not only has the bilingualism been successful, I believe that it has provided many more cognitive benefits such as adaptability and good listening skills.’*

*‘I do not want to limit my child's opportunities just because he has DS.’*

There was also reference to differences across countries and the bilingual context. For example, rather than reducing language input to one language, some families had chosen to limit exposure to two languages as opposed to three.

*‘We live in Switzerland where everyone is bi or multilingual (my husband speaks 4‐5 languages!). The decision here wasn't to stick to only one language, it was to stick to two and not three.’*

*‘Our original plan was for my husband to speak Catalan AND Spanish to our son, and for me to speak English. After discussing it we decided to stick to Spanish and English only.’*

Other parents stated that they've taken a more flexible approach to bilingualism and that it may be introduced at a later time but still with the intention for their children to be bilingual.

*‘Childhood is a long time, perhaps especially with a child with DS, and things change over the years in terms of what's possible; I haven't given up on bilingualism for my child with DS but it might look like that currently.’*

*‘English is our main focus because we live in England and our daughter will grow up here, but eventually when she becomes a little older and is a little more vocal, we will try to introduce a second language, but for now, English is our main concern because her future (e.g. schooling) will be in England.’*

#### 3.9.2 Challenges and Language Outcomes

Despite this, a couple of parents expressed ongoing concerns about bilingualism felt more conflicted about raising their children bilingually.

‘*I feel that my child's ability to express herself would have been a lot better if we (family, school professionals and therapists) focused only on one language.’*

*‘He has some language delay (a few words in each language only) and people frequently suggest this is due to bilingualism, but I am not sure that this reflects research. I would be interested to see if this is the case*.’

At the same time, other parents reported that bilingualism had been successful for their families.

*‘Considering all the barriers… [she] still managed to learn the family language at her own pace and successfully achieved her language goals. Another thing we have noticed is that she could easily picked up other languages other than English and Italian. Showing her brain is adapted to new way of learning.’*

*‘*My child who has Down Syndrome understands Punjabi very well even though she can't speak it very well… it's very rewarding and good to know how much it will benefit her in the future.’

*‘Her understanding is good in both languages as the nursery works very hard with her.’*

The limited understanding around the developmental outcomes for children with DS raised in bilingual environments was also highlighted in these reflections.

*‘I would have liked to know a bit more about the advantages and disadvantages of learning two languages… Is there a speech delay (further) due to hearing two languages at home?’*

#### 3.9.3 Use of Sign Language

Many families reported how they had incorporated sign language or Makaton^2^, to aid communication, especially with younger non/pre‐verbal or partially verbal children. This was reported to specifically help bridge the gap between the two languages they the children were exposed to. Sign language was considered important for these families, and it gave the children a way to communicate even before they were fully verbal.

*‘I think that use of Makaton helped my daughter in learning two languages. As we signed as we spoke and initially she only signed.’*

‘*Makaton has played a big part in communication with our daughter. She has started to babble a little bit but she knows a lot of signs and she will use these independently too without prompting. I wish nurseries and schools would include Makaton/BSL in their curriculum to make it a more universal form of communication for those who are not vocal or cannot speak English’*

#### 3.9.4 Support Systems and Professional Guidance

Finally, several reflections from families expressed how professional advice, or lack thereof, had shaped language decisions for children with DS. Some even reported hesitancy in seeking guidance on bilingualism, fearing negative judgment from professionals.

‘*I don't tend to mention bilingualism much to professionals as I feel they might judge us that we are making our daughters life harder by attempting bilingualism.’*

*‘The support service available for children with Down Syndrome encouraged the learning of one language i.e., English… I was happy and in agreement with this.’*

*‘There is no advice and support’*

In stark contrast to this, some families were supported in raising their children bilingually. For some families, this positive approach had a strong impact and made them feel supported in their decision, whilst others continued to receive mixed support but continued with bilingualism regardless.

‘*We were advised to use both languages parallel and it works.’*

*‘Almost everyone who supports our child with Down's Syndrome has encouraged us to bring him up bilingually, although his main speech and language therapist (who speaks only English) has advised us not to do this and to choose “one language” as bilingualism is going to confuse him. When we said Welsh would be the language we would choose if we had to choose, they were critical of our choice.’*

## 4 Discussion

This study aimed to provide a novel insight into the experiences of bilingual families raising children with DS. Results highlighting both the positive attitudes parents hold toward bilingualism and the persistent challenges they face. Consistent with recent research (e.g., Kay‐Raining Bird et al. 2012; Drysdale 2015), our findings suggest that parents have significant concerns about bilingualism, though they may still choose to raise their child bilingually, often motivated by cultural identity, community belonging, and perceived cognitive and social advantages. Despite concerns, the majority observed expected bilingual language development patterns in their children, reinforcing the view that bilingualism can be both feasible and beneficial in this population.

However, a central theme emerging from this study is the variability and often inconsistency in professional guidance. Although some parents received supportive and informed advice—particularly from SLTs—others encountered mixed messages or discouragement from professionals such as health visitors, social workers, and educational staff. These findings echo previous research showing that parents of children with developmental disabilities often receive conflicting messages about bilingualism, leading to confusion and hesitancy about language choices (Howard et al. 2021; Yu 2013; Jegatheesan 2011). Importantly, some families reported receiving contradictory advice from different practitioners, which may increase the likelihood of uncertainty, hesitation, or anxiety when making decisions about their child's language development.

The finding that families receiving positive professional guidance were more likely to persist in promoting bilingualism highlights the critical role that practitioners play in shaping parental decisions. This aligns with recent studies emphasising the influence of professional attitudes on family language practices in developmental contexts (Gonzalez‐Barrero and Nadig 2019). When advice is grounded in current evidence and delivered confidently, it can encourage families to maintain home languages and engage in bilingual opportunities. Despite growing awareness among SLTs about the feasibility of bilingualism in these populations, this study reveals substantial inconsistency in the advice offered across other professional domains. For example, some GPs or paediatricians seemed less confident to provide guidance on bilingualism, whilst at the same time, many others were supportive of bilingualism. This highlights the potential inconsistency amongst professions which could impact parental decision making.

The concerns raised by over half of participating parents, such as language confusion, difficulty acquiring two languages, and lack of systemic support, reflect a broader need for clearer and more accessible resources. Although recent research continues to counter the myth that bilingualism is detrimental to children with DS or other neurodevelopmental conditions (Paradis and Govindarajan 2018; Peña et al. 2023; Ward et al. 2021), families still encounter structural barriers, such as a shortage of bilingual therapists and a lack of culturally and linguistically responsive education provision. These challenges can reinforce perceptions that bilingualism is a ‘risk’, particularly among families navigating complex developmental needs.

### 4.1 Implications on Clinical Practice and Future Research

In the UK, professional guidelines exist in relation to bilingualism from The Royal College of Speech and Language Therapists (RCSLT). These stipulate that bilingualism does not contribute to speech, language, or communication disorders and should be actively supported in clinical practice (RCSLT 2020). In addition, these clinical guidelines encourage professionals to ‘provide advice on maintaining bilingualism by encouraging home language use’ to foster cognitive flexibility, social engagement, and cultural identity (Pert and Bradley 2018). This should be followed ‘even when individuals have very limited communication,’ which may be the case for some children with DS, particularly during the early years.

Despite these recommendations, parental reports in this study indicate inconsistencies in professional advice. A substantial proportion of parents did not receive any professional guidance regarding bilingualism, which may have left them feeling uncertain about how best to support their child's language development. Encouragingly, our findings indicate a potential shift in professional attitudes as parents of younger children reported more supportive guidance compared to those whose children are older. Whilst the impact of age was seen, this was small and not statistically significant, however, may reflect increased dissemination of bilingualism research and evolving professional training, particularly in speech and language therapy (RCSLT 2021). Nevertheless, this shift appears uneven and highlights the importance of standardising professional knowledge across sectors. Without clear, consistent, and coordinated messages, families are left to make complex decisions without adequate support. These decisions can have long‐term implications for their child's identity, social connectedness, and educational access.

Additionally, this uncertainty around bilingualism appeared to decrease when parents engage in discussions with professionals, suggesting that informed advice plays a key role in supporting bilingual families. Future research should prioritise the perspectives of professionals themselves, exploring their training, beliefs, and confidence in advising bilingual families. It will also be important to extend this work into diverse linguistic and cultural settings, and to include longitudinal data to better understand language trajectories over time in children with DS. Integrating bilingualism into standard care pathways, developing professional development materials, and co‐producing resources with families may all contribute to more inclusive and equitable support.

### 4.2 Limitations

Although this study offers valuable insights into parental perspectives on bilingualism, several limitations must be acknowledged. First, there is a likely self‐selection bias in the participant pool. Those who chose to take part may have had a particular interest in, or positive experiences with, bilingualism. As a result, their perspectives may not fully represent families who encountered more neutral or negative experiences or who disengaged from bilingualism altogether. Therefore, whilst some respondents report more favourable experiences and advice supporting bilingualism, these may not be representative of all parents’ experiences. These parents are also likely to be more highly educated than the general population, although this was not explored as part of the study. Second, the study relied on retrospective accounts, meaning that participants were recalling experiences, beliefs, and advice they received. Memory is inherently selective, and perceptions may have evolved over time, particularly as children have grown, and language trajectories have unfolded. These experiences therefore reflect the advice and support that they received at different time points, and families in similar positions may now be receiving more evidence‐informed guidance, though further research is needed to evaluate this. Nevertheless, such reflections provide valuable insights into the long‐term resonance and perceived impact of early advice. Third, while participants came from a range of geographic and policy contexts, we believe that even within the countries included in the study, there may be meaningful differences depending on local language environments. For example, individuals living in multilingually rich areas such as London may have encountered more supportive attitudes and institutional resources than those in regions where bilingualism is less visible or less actively promoted. Additionally, different parts of the UK operate under distinct language policies, which may shape the kind of advice professionals are legally or ethically permitted to give. For instance, in Wales, public services are legally required to treat Welsh no less favourably than English (Welsh Language Act 1993), meaning that professionals may not have been permitted to advise against the use of Welsh, even if such advice might occur in other contexts. These regional and policy‐based differences complicate direct comparison across responses, and point to the need for more nuanced, context‐sensitive research, especially in areas where bilingualism lacks formal support.

## 5 Conclusion

Our study suggests that parents and caregivers raising children with DS bilingually often experience uncertainty and concern about the effects of dual language exposure. Although bilingualism was generally viewed as valuable by the parents in the present study, it was often perceived as more appropriate or attainable for siblings without DS. Interestingly, our findings indicate that the broader societal support for bilingualism, such as in contexts like England where such support is limited or non‐existent, does not appear to significantly modulate parental beliefs, practitioner advice, or reported success in bilingual language development. However, this observation should be interpreted with caution, as a more in‐depth and context‐sensitive investigation is needed to confirm and better understand this trend. Furthermore, the advice parents receive about bilingualism and DS varies widely depending on the professional background of practitioners and is frequently conflicting. This highlights an urgent need for further research into how practitioners’ training, professional roles, and personal beliefs shape the guidance they provide to families. Analysis of qualitative responses reveals a complex landscape of views, with respondents expressing a wide range of emotions, beliefs, and experiences, often shaped by inconsistent and, at times, non‐evidence‐based information. Understanding these dynamics is crucial for developing more coherent and informed support for families navigating bilingualism in the context of DS.

## Supporting information




**Supporting Information**: jlcd70153‐sup‐0001‐SuppMat.docx

## Endnotes

1 Here we use the term ‘*bilingual’* to refer to someone who speaks two or more languages. We use this in an inclusive way to embrace the diversity of bilingual and multilingual individuals, acknowledging the wide range of experiences and language use across different contexts.

2 Makaton is a communication programme that combines speech with signs (based on sign language) and symbols to support people with speech, language, or communication difficulties. It is widely used with children and adults who have additional needs or learning disabilities. For more information, see https://makaton.org/
