# Supplementary material for: Attitudes Towards Bilingualism: Insights From Parents of Children With Down Syndrome
Source: Int J Lang Commun Disord. 2025 Nov 8;60(6):e70153. doi: 10.1111/1460-6984.70153 (PMC12595590; doi:10.1111/1460-6984.70153)
Supplement: Supplementary file 1 — Supporting Information: jlcd70153‐sup‐0001‐SuppMat.docx [file JLCD-60-0-s001.docx]

Respondent information:

1. Name:
2. Email:
3. Age:
4. Relationship to the child: mother, father, other

Child information:

1. Child’s age in years and months:
2. Does your child have a diagnosis of Down Syndrome?
   1. Yes, No
3. If your child has a diagnosis of Down syndrome, are you aware if they have mosaic or translation subtypes?
   1. Yes – mosaic, Yes – translocation, No – not aware, No – Full Trisomy 21
4. Does your child have any other diagnosis other than Down syndrome?
   1. Yes, No
5. If your child does have another diagnosis, please specify it here:
6. Does your child have a hearing disorder?
   1. Yes, No
7. If yes, has this been corrected in any way (e.g., use of hearing aids)?
   1. Yes, No

Caregiver’s information (your information):

1. Relationship to the child: mother, father, other
2. Your age:
3. Your nationality:
4. Country you were born:
5. If you were not born in the UK, where were you born?
6. If you were not born in the UK, how old were you when you moved to the UK?
7. What is your mother tongue (i.e., the language your primary caregivers spoke to you when you were a child)?
8. What is your highest level of education?
   1. GCSE’s, Apprenticeship, A level, Bachelor’s degree, Master’s level degree, doctoral degree, other.
9. Are you employed? Yes/No
10. If yes, what type of work do you do?
11. Rate your language abilities in English:
    1. How well do you speak English:
    2. How well do you write English:
    3. How well do you read English:
    4. How well do you understand English:
12. Rate your language abilities in your mother tongue?
    1. How well do you speak this language:
    2. How well do you write this language:
    3. How well do you read this language:
    4. How well do you understand this language:

Caregiver’s B information (if there is another main caregiver in the household):

1. Relationship to the child: mother, father, other
2. Caregiver B’s age:
3. Caregiver B’s nationality:
4. Country they were born:
5. If they were not born in the UK, where were they born?
6. If they were not born in the UK, how old were they when you moved to the UK?
7. What is their mother tongue?
8. What is their highest level of education?
   1. GCSE’s, Apprenticeship, A level, Bachelor’s degree, Master’s level degree, doctoral degree, other.
9. Are they employed?
10. If so, what type of work do they do?
11. Rate their linguistic abilities in English:
    1. How well do you speak English:
    2. How well do you write English:
    3. How well do you read English:
    4. How well do you understand English:

Other information

1. Where do you live?
2. What is the estimate family gross annual income?

Language information:

1. Is there more than one home language in your household? Yes/No
2. What is/are the home language(s):
3. What is the main home language?
4. At what age was your child exposed to English?
5. Please, can you provide an estimation of how many hours your child is exposed to English:
   1. at home during weekdays?
   2. outside the home environment during weekdays?
   3. at home during weekends?
   4. Outside the home environment during weekends?
6. Please, rate the overall expressive speech (production) of your child in English?
7. Please, rate the overall receptive speech (comprehension) of your child in English?
8. At what age was your child exposed to the other language?
9. Please, can you provide an estimation of how many hours your child is exposed to the other language:
   1. at home during weekdays?
   2. outside the home environment during weekdays?
   3. at home during weekends?
   4. Outside the home environment during weekends?
10. Please, rate the overall expressive speech (production) of your child in the other language?
11. Please, rate the overall receptive speech (comprehension) of your child in the other language?

**Questionnaire: Feelings about bilingualism**

1. Before your child was born, did you intend to use more than one language with them?
2. Did their diagnosis of DS change the way you speak with your child or other children?
3. How important is it to you for your child to become bilingual on a scale from 1 to 10?
4. How important is it to you for child/children without DS in your family to become bilingual, compared to your child with DS on a scale from 1 to 10? (Skip this question if you have no other children without DS).
5. If you believe bilingualism is an important goal for your child with DS, please rank the 3 most important reasons that apply to you:
   1. Communication with family members
   2. Communication with people in your neighborhood
   3. Communication with people in school
   4. I live in a bilingual/multilingual city
   5. I live in a bilingual/multilingual country
   6. Religious and/or cultural activities
   7. Provides more life opportunities
   8. Important in the job market
   9. Other (please specify)
6. Do you have concerns about helping your child with DS to become bilingual? YES/NO
7. If yes, please indicate why you are concerned about your child becoming bilingual. Rank the 3 most important reasons that apply to you: 1 = most important, 2 = second most important, 3 = third most important.
   1. Learning 2 languages is too hard for my child
   2. There is little or no professional help for this
   3. I cannot help my child learn another language
   4. I am afraid my child will be confused by two languages
   5. I do not have access to services that would help me or my child with bilingualism
   6. My family and/or friends will not support my decision
   7. Other (please specify)
8. What choice have you made for your child with DS? (please indicate only one)
   1. I have chosen for my child with DS to learn one language only __________
   2. I have chosen for my child with DS to learn 2 (or more) languages ___________
9. If you have chosen for your child with Down Syndrome to learn 2 (or more) languages, please use ‘x’ to indicate all the strategies you currently use. If a strategy does not apply to you, leave it blank.
   1. My child has a language tutor
   2. My child takes special language classes
   3. My child gets bilingual speech–language pathology support
   4. We teach our child language skills at home
   5. Different people speak different languages at home
   6. Different languages are spoken at different times at home
   7. My child attends preschool/school in a second language
   8. Watch TV in 2 (or more) languages
   9. Read books in 2 (or more) languages
   10. Other (please specify)
10. If you have chosen for your child with Down Syndrome to learn 2 (or more) languages, please use ‘x’ to indicate any challenges that you have come across. If you don’t feel that any of these challenges apply to you, leave it blank.
    1. There is not enough provision available to support my child
    2. I do not know how to support my child bilingually
    3. There is not enough speech-language therapy provision
    4. I am not bilingual myself
    5. There was not enough information available about bilingualism
    6. Other (please specify)
11. If you have chosen for your child with DS to learn 2 (or more) languages, how would you rate your success in helping your child with DS learn 2 (or more) languages?
    1. Scale: 1 = not at all successful, 2 = somewhat, 3 = successful, 4 = very successful, 5 = extremely successful
12. If you have a child/other children without Down Syndrome and you decided for them to learn 2 (or more) languages, please use ‘x’ to indicate all the strategies you currently use. If a strategy does not apply to you, leave it blank.
    1. My child has a language tutor
    2. My child takes special language classes
    3. My child gets bilingual speech–language pathology support
    4. We teach our child language skills at home
    5. Different people speak different languages at home
    6. Different languages are spoken at different times at home
    7. My child attends preschool/school in a second language
    8. Watch TV in 2 (or more) languages
    9. Read books in 2 (or more) languages
    10. Other (please specify)
13. If you have a child/other children without Down Syndrome and you decided for them to learn 2 (or more) languages, please use ‘x’ to indicate any challenges that you have come across. If you don’t feel that any of these challenges apply to you, leave it blank.
    1. There is not enough provision available to support my child
    2. I do not know how to support my child bilingually
    3. There is not enough speech-language therapy provision
    4. I am not bilingual myself
    5. There was not enough information available about bilingualism
    6. Other (please specify)
14. If you have chosen for your child/other children without Down Syndrome to learn 2 (or more) languages, how would you rate your success in helping your child without DS learn 2 (or more) languages?
    1. Scale: 1 = not at all successful, 2 = somewhat, 3 = successful, 4 = very successful, 5 = extremely successful

**ADVICE**

1. Have you ever received any professional advice regarding bilingual language exposure for your child? Yes, No, Other
2. If you have received advice, who gave it to you?
   1. Medical staff, Nursery/primary/secondary education staff, Speech and Language Therapist, Other:
3. If you have received advice, which of the following statements best captures it:
   1. I was encouraged to promote the home language with the child.
   2. I was discouraged to promote the home language with the child because it might be detrimental for their expressive abilities in English
   3. I received mixed information about bilingualism
   4. I did not receive any information about bilingualism
   5. Other:

**ADVICE**:

Professionals sometimes express opinions about bilingualism and children with DS. Please indicate what advice each professional have given you selecting all that apply. If a professional has not advised you in this area, please leave blank.

1. Family Physician (select all that apply):
   1. No, do not raise your child bilingually
   2. Yes, raise your child bilingually
   3. No advice was given
   4. Use each language with different people
   5. Use each language in different places (e.g., home, school)
   6. Use each language at different times
   7. Other (specify):
2. Speech–Language Pathologist (select all that apply):
   1. No, do not raise your child bilingually
   2. Yes, raise your child bilingually
   3. No advice was given
   4. Use each language with different people
   5. Use each language in different places (e.g., home, school)
   6. Use each language at different times
   7. Other (specify):
3. Psychologist (select all that apply):
   1. No, do not raise your child bilingually
   2. Yes, raise your child bilingually
   3. No advice was given
   4. Use each language with different people
   5. Use each language in different places (e.g., home, school)
   6. Use each language at different times
   7. Other (specify):
4. Social Worker (select all that apply):
   1. No, do not raise your child bilingually
   2. Yes, raise your child bilingually
   3. No advice was given
   4. Use each language with different people
   5. Use each language in different places (e.g., home, school)
   6. Use each language at different times
   7. Other (specify):
5. Classroom/preschool teachers (select all that apply):
   1. No, do not raise your child bilingually
   2. Yes, raise your child bilingually
   3. No advice was given
   4. Use each language with different people
   5. Use each language in different places (e.g., home, school)
   6. Use each language at different times
   7. Other (specify):
6. Do you feel the covid pandemic affected any of your linguistic choices?

Yes, No

1. If yes, how?

Thank you for taking the time to respond. Your responses are greatly valued.
